# Un essai contrôlé randomisé: effet du port de chaussures à talons hauts sur le squelette appendiculaire inferieur

**DOI:** 10.11604/pamj.2015.20.191.4495

**Published:** 2015-03-02

**Authors:** Fifamè Eudia Nadège Koussihouèdé, Jean-Marie Falola, El-Mansour Barres Fousseni

**Affiliations:** 1 Laboratoire de Biomécanique et Performance (Labiop), Institut National de la Jeunesse, de l'Education Physique et du Sport (INJEPS), Porto-Novo, Benin; 2 Laboratory of Human Motricity, Education Sport and Health (LAMHESS), Unité de Formation et de Recherche en Activité Physique et Sportive, Université du Sud, France

**Keywords:** Posture, chevilles, genoux, chaussures à talons, Posture, ankles, knees, high heels shoes

## Abstract

**Introduction:**

Véritable attribut de la féminité, le port de chaussures à talons est devenu une exigence professionnelle dans certaines structures des pays en développement comme le Bénin. L'objectif de ce travail est de déterminer les effets spécifiques du port de chaussures à talons hauts sur le squelette appendiculaire inférieur.

**Méthodes:**

Des examens radiographiques de face et de profil de la cheville et du genou ont été effectués sur 122 femmes volontaires, âgées en moyenne de 25,09 ± 1,34 ans et ayant les genu varum qui ont participé à cette étude.

**Résultats:**

Les résultats ont indiqué une augmentation significative de l’écart inter malléolaire, de l’écart inter condylien, de l'angle antérieur du pied et de l'angle fémoro-patellaire. Une diminution significative de l'angle postérieur du pied a été constatée.

**Conclusion:**

Le port de chaussures à talons hauts est l'une des causes de la gonarthrose, des troubles des articulations de la cheville et du pied.

## Introduction

Déplacer son corps, en totalité ou en partie, est une activité humaine familière, dont la pratique offre notamment de saisissants exemples [1]. Dans la réalisation de cette tâche, la difficulté consistera à se mouvoir tout en conservant l’équilibre postural. Ainsi, Massion [2] parle de fonction d’équilibration qui consiste à maintenir l’équilibre tant au cours des mouvements qu'en posture debout. Cette posture de référence nommée par Paillard [3] comme la posture érigée fondamentale est définie selon Winter [4] comme l'orientation de l'ensemble des segments du corps d'un sujet par rapport à la verticale gravitationnelle. Le contrôle de la posture debout a de ce fait été modélisé comme un pendule inversé oscillant autour d'une position fixe [4]. Dans ce modèle, le corps oscille autour de l'axe de rotation de la cheville et son support est le polygone de sustentation constitué par les empreintes deux pieds du sujet. Dans la majorité des études posturographiques, les sujets sont placés pieds nus sur une plate forme de force. Menz et Lord [5] ont par exemple étudié l'effet du port de chaussures sur la stabilité posturale des personnes âgées. Cette recherche a indiqué que toute modification survenant entre la plante de pied et le sol est susceptible d'affecter la posture. Prévues pour protéger les pieds, toutes les chaussures ne présentent pas les mêmes caractéristiques en termes de prévention des chutes [5–7]. La caractéristique la plus importante mise en cause est la hauteur du talon de la chaussure. Or l'accomplissement des tâches quotidiennes et professionnelles impose le port de chaussures. En Afrique subsaharienne et particulièrement au Bénin, le constat est que le port de chaussures à talons hauts est un comportement vestimentaire qui est adopté par la majorité des femmes dans la réalisation des tâches professionnelles. De ce fait, elles sont très exposées aux effets délétères de ce comportement vestimentaire. Dans la littérature, Snow et Williams [8] ont rapporté que contrairement à la condition pieds nus, la répartition de la masse corporelle est modifiée en chaussures à talons hauts et que l'avant-pied, malgré sa faiblesse supporte les 2/3 de la masse corporelle en posture debout. D'autres recherches [9, 10] ont montré que le port de chaussures à talons hauts augmente le risque de chute et de glissade chez les sujets. Au plan ostéologique, l'impact du port de chaussures à talons hauts sur les articulations en posture debout n'a pas été étudié. L'objectif de ce travail est de déterminer les effets spécifiques du port de chaussures à talons hauts sur le squelette appendiculaire inférieur en comparant les angulations articulaires des membres inférieurs et les écarts inter osseux des femmes béninoises avec et sans chaussures à talons hauts.

## Méthodes

**Type d’étude et cadre de réalisation:** il s'agit d'une étude prospective contrôlée, réalisée dans les trois villes à statut particulier de la République du Bénin de Novembre 2012 à Janvier 2013. Cette étude a eu pour cadre le Laboratoire de Biomécanique et Performance de l'Institut National de la Jeunesse, de l'Education Physique et du Sport puis le Service d'Imagerie Médicale du Centre Hospitalier de Pneumo-Phtisiologie de Porto-Novo. Celui-ci est le centre de référence en radiologie au Bénin; c'est la raison pour laquelle toutes les femmes incluses dans l’étude y ont subi les examens radiographiques.

**Population d’étude et échantillonnage:** la population cible est celle des femmes porteuses de chaussures à talons hauts des services et institutions financières publics et privés des trois villes à statut particulier du Bénin. L’étude a été réalisée avec un échantillon non probabiliste de 122 femmes volontaires ayant les genu varum, âgées de 25,09 ± 1,34 ans. Etaient inclus dans cette étude, les femmes fonctionnaires, ayant le port de talons comme exigence professionnelle et qui n'avaient aucun handicap moteur.

**Variables mesurées:** les mesures de la masse corporelle et de la taille ont été effectuées en utilisant respectivement un pèse personne (Seca) précis à 0,5 kg près puis un bodymètre Seca 206, de portée 2,20 m, précis à 1 mm près. Les angles antérieur et postérieur du pied, l'angle fémoro-patellaire ont été mesurés sur les clichés issus des examens radiographiques. Les écarts inter malléolaire et inter condylien ont été aussi mesurés.

**Procédures:** toutes les mesures et examens radiographiques ont été effectués de façon randomisée sur les sujets en posture debout, dans les conditions pieds nus et port de chaussures à talons de hauteur moyenne de 8,1 ± 2,1 cm. Les angulations articulaires ont été mesurées sur les films radiographiques avec le goniomètre Dela de marque Rostfrei de portée 180° en utilisant les repères anatomiques. L'angle antérieur du pied (AAP) est recueilli sur la face antérieure du membre inférieur au niveau de l'intersection des segments (épicondyle latéral du genou - apex de la malléole latérale) et (apex de la malléole latérale - phalange distale du petit orteil); l'angle postérieur du pied (APP) a été mesuré au niveau des segments sécants de (l’épicondyle latéral du genou - apex de la malléole latérale) et (apex de la malléole latérale - tubérosité postérieure du calcanéum); l'angle fémoro-patelaire (AFP), encore appelé angle Q a été obtenu par l'intersection de la ligne provenant de l’épine iliaque antéro-supérieure et celle passant par l'axe médian de la patella. Il a été mesuré selon la technique de Smith et al [11]; l’écart inter malléolaire (EIM) et l’écart inter condylien (EIC) ont été mesurés selon la méthode de Samaei et al [12]; un appareil, à rayon X de marque Apelem GTI 50 Hz et de type 400 V ± 10% a été utilisé pour les examens radiographiques par un médecin, spécialiste en imagerie médicale. De grandes cassettes format 30 x 90 cm ont été utilisées pour les examens et la même distance foyer-film (1 m) a été respectée pour tous les sujets.

**Analyse des données:** la moyenne et l’écart-type ont été calculés pour chaque variable. Une ANOVA à un facteur a permis de déterminer l'effet du port de chaussures à talons hauts sur toutes les variables mesurées. Le test post hoc de Turkey a permis d'identifier les différences significatives. Le seuil de signification pour toutes les analyses est fixé à p < 0,05.

**Considérations éthiques:** une séance préalable d'information et de sensibilisation des sujets a été organisée en vue d'expliquer les objectifs, l'intérêt, le protocole de l’étude, le gain immédiat et les risques minimes que les sujets encourent en participant à l’étude, avant de donner leur consentement éclairé et écrit. Cette séance a eu lieu dans les institutions identifiées, où le port de talons hauts est une exigence vestimentaire. Avant que ne soit effectuée la prise des données, le protocole de l’étude a reçu l'approbation du Comité Scientifique Sectoriel des Sciences et Techniques des Activités Physiques et Sportives de l'Université d'Abomey-Calavi d'une part et celle du Comité National Provisoire d'Ethique de la Recherche en Santé d'autre part.

## Résultats

**Figure 1:**
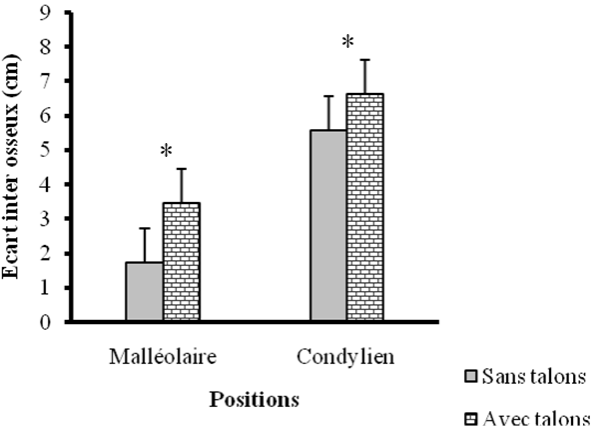
Variation des angles antérieur et postérieur du pied puis de l'angle fémoro-patellaire avec et sans chaussures à talons

**Figure 2:**
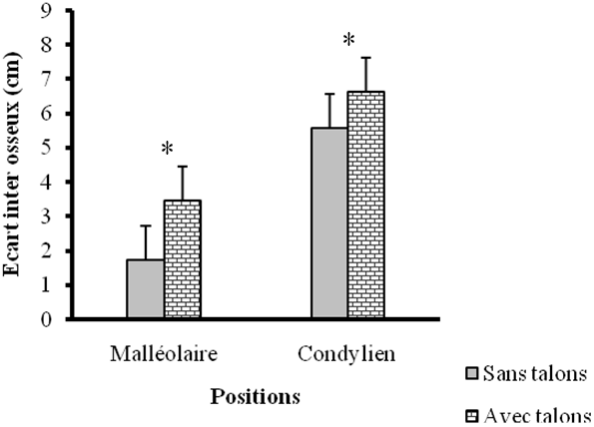
Variation des écarts inter malléolaire et condylien avec et sans chaussures à talons

**Figure 3:**
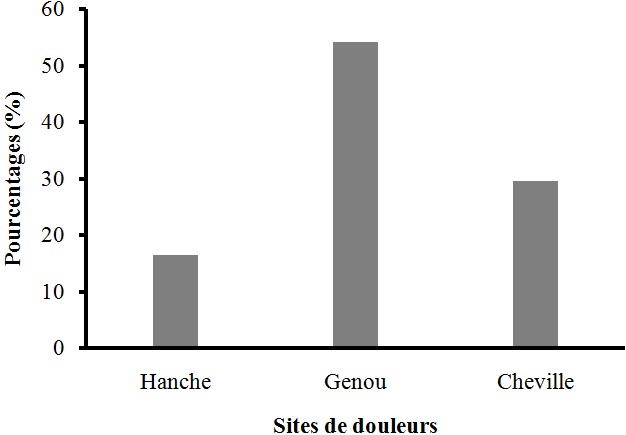
Variation des pourcentages des sites de douleurs des sujets en chaussures à talons

Les caractéristiques des sujets (Tableau 1) de cette étude ont montré qu'il s'agissait d'une population jeune d'un âge moyen de 25,09 ± 1,34 ans. Elles ont indiqué, compte tenu de l'indice de masse corporelle (IMC moyen = 23,68 kg/m2 ± 0,32) que l’échantillon d’étude est composé de femmes normo-pondérées. Sous les deux conditions (pieds nus et avec talons hauts) les résultats des angulations articulaires (Figure 1) ont révélé au niveau de la cheville, une augmentation significative de l'angle antérieur du pied (AAP), contre une diminution significative de l'angle postérieur du pied (APP). Quant au genou, une augmentation significative de l'angle fémoro patellaire (AFP) due au port de talons hauts a été enregistrée. L'observation des écarts inter malléolaire (EIM) et inter condylien (EIC) (Figure 2) a montré une augmentation significative entre la condition pieds nus et celle avec talons hauts. Les douleurs ressenties par les sujets et qui sont dues au port de chaussures à talons hauts étaient accentuées au genou et à la cheville (Figure 3).

**Tableau 1:** Caractéristiques biométriques des sujets

Moyenne ± écart type (n = 122)	
Age (ans)	25,09 ± 1,34
Taille (m)	1,64 ± 0,65
Masse corporelle (kg)	64,20 ± 1,70
Indice de masse corporelle (kg/m^2^)	23,68 ± 0,32

## Discussion

Les résultats importants de cette étude indiquent qu'il y a un effet remarquable du port de chaussures à talons hauts sur les articulations des membres inférieurs. L'analyse des clichés radiographiques de l’échantillon d’étude a pris en compte plusieurs paramètres qui ont été observés au plan ostéologique grâce aux repères osseux. Les variations observées au niveau des angulations articulaires indiquent que l'angle antérieur du pied (AAP) et l'angle postérieur du pied (APP) (Figure 1) fonctionnent de façon agoniste et antagoniste car l'augmentation significative (p < 0,05 pour AAP) de l'un, entraîne la diminution significative (p < 0,05 pour APP) de l'autre. En effet, ceci peut se rapporter dans le cycle de marche, au mouvement de la cheville en fin de phase d'appui où le talon du pied arrière est soulevé du sol et seul son avant-pied est en contact avec celui-ci; inversement, le talon du pied avancé est en contact avec le sol et l'avant-pied de ce dernier est décollé de la surface de marche [13]. Parallèlement, le tibial antérieur et le triceps sural qui sont les principaux muscles de la jambe sollicités lors de la marche [14], sont contractés de façon alternative et synergique afin de favoriser le fonctionnement agoniste et antagoniste des angles antérieur et postérieur du pied lors du port de chaussures à talons hauts. Notons que dans le cas du cycle de marche, cette phase ne dure que quelques millisecondes, alors qu'en posture debout, la cheville est maintenue dans cette position pendant toute la durée du port de chaussures à talons hauts. L'augmentation significative observée au niveau de l'AAP, encore appelé angle de la dorsiflexion, a conduit dans ce contexte à un étirement des tendons et ligaments dans le plan antérieur, ce qui est source de tensions musculaires et de douleurs podales [15].

Par contre dans le plan postérieur, la diminution significative de l'APP réduit la longueur du tendon d'Achille et a entraîné la rigidité de ce dernier [16]. C'est ce qui explique la tonicité musculaire du gastrocnémien observée chez les femmes porteuses de chaussures à talons en permanence [17]. Ce fonctionnement alterné des angles antérieur et postérieur du pied (AAP et APP), a pour conséquence la modification de la répartition de la masse corporelle. En effet, en posture debout pieds nus, les deux tiers (2/3) de la masse corporelle sont supportés par le talon du pied et le tiers (1/3) par les orteils [8]. En chaussures à talons hauts, les forces d'impact appliquées à l'avant-pied augmentent et font déplacer le corps dans le plan antérieur [8] ce qui conduit à l'ouverture de l'angle antérieur du pied (AAP). La mesure de l'angle antérieur du pied (AAP) et celle de l'angle postérieur du pied (APP) étant effectuées sur des plans opposés, l'augmentation de l'un entraîne la diminution de l'autre; ce qui explique la diminution de l'angle postérieur du pied (APP) en chaussures à talons hauts. Ces modifications angulaires ne sont pas sans effets sur l’écart inter malléolaire (EIM) (Figure 2) qui a subi une augmentation significative en chaussures à talons hauts. Cette augmentation est due aux changements observés pour AAP et APP qui sont mesurés au niveau de la cheville. Bien que cet écart ait subi une augmentation significative, celle-ci n'a pas été suffisante pour induire un valgum (EIM > 3 cm) [12]. Toutes ces variations observées au niveau du pied constituent la cause des douleurs podales dont se sont plaints nos sujets. Ce résultat est en conformité avec celui de Wegener et al [15] qui ont indiqué que le port de chaussures à talons hauts peut causer des oignons, des cors, des callosités plantaires, le hallux valgus etc. puisque l'avant-pied constitue la partie faible du pied. De plus, en chaussures à talons hauts, toute la plante de pied n'est pas en contact avec le sol; le polygone de sustentation est de ce fait réduit, le point de projection du centre de gravité et le centre de pression se trouvent alors modifiés [18].

Nos résultats indiquent au niveau du genou, une augmentation significative de l'angle fémoro-patellaire (AFP) encore appelé angle Q. Cette augmentation provoquera une tension au niveau du ligament patellaire en l’étirant; ce qui va réduire les mouvements au niveau du genou. C'est ce qui explique la diminution de l'angle de flexion-extension du genou à la phase d'oscillation pendant la marche en chaussures à talons hauts [8, 19]. Notre résultat va dans le même sens que celui de Park et al [20] qui ont indiqué qu'en chaussures à talons hauts, un genou reçoit très souvent une norme disproportionnée de force par rapport à l'autre genou, ce qui provoque des subluxations. Ces risques de blessures sont confirmés par Kerrigan et al [21] qui ont trouvé de pareils résultats en mesurant, en chaussures à talons hauts, le moment des forces des sujets à genu varum. Cette augmentation significative constatée a conduit inévitablement à une augmentation des forces de compression dans le compartiment interne du genou. Ils ont conclu que, soumettre le genou à un tel stress conduirait alors à des conséquences destructrices. Les résultats de notre étude indiquent une augmentation de l’écart inter condylien (EIC) (Figure 2). En effet, les sujets de notre étude ayant au départ les genu varum (EIC > 3 cm) ont connu une accentuation de l’état varum de leur genou en chaussures à talons hauts [12]. L'augmentation de l’écart inter condylien pourrait se comprendre par un glissement dans le plan latéral de la tête tibiale. Les modifications enregistrées au niveau de l'angle fémoro-patellaire et de l′écart inter condylien montrent que le genou est fortement affecté par le port de chaussures à talons hauts. Ces changements observés ont pour conséquence un mauvais positionnement des têtes articulaires fémorale, tibiale et patellaire, provoquant ainsi leur usure rapide. Selon Cowley et al, [19] ce phénomène conduit à la gonarthrose. Elle est davantage justifiée par les douleurs au genou et à la cheville dont se sont plaints nos sujets.

Les résultats de notre étude révèlent un effet significatif du port de chaussures à talons hauts sur les articulations des membres inférieurs. Le port de chaussures à talons hauts peur être considéré comme facteur favorisant de la gonarthrose, des vingt neuf troubles des articulations de la cheville et du pied. Les limites de notre étude résident dans la prise en compte des sujets normo pondérés et n'ayant que des genu varum. Une étude réalisée sur les femmes obèses et/ou ayant une autre forme de genoux nous fournirait plus d'informations puisque le port de chaussures à talons hauts ne faisait pas partie de la culture africaine. Dans ce contexte les effets du port de chaussures à talons hauts sur la marche chez des femmes en Afrique se révèle d'une importance capitale.

## Conclusion

Les modifications posturales dues au port de chaussures à talons hauts sont enregistrées au niveau de toutes les articulations étudiées. Les changements observés sur les angulations articulaires affectent la posture du membre inférieur, ce qui amène le sujet à s'incliner, faisant ainsi déplacer la ligne de gravité vers l'avant. Outre les nombreux changements posturaux observés dans cette étude, il est à noter que la forme du genou est modifiée avec le port de chaussures à talons hauts. Au vue des résultats pertinents de cette étude, l'approfondissement des recherches sur le phénomène du port de chaussures à talons hauts se révèle d'une importance capitale en milieu professionnel dans le contexte Africain.
